# Development of Portable Digital Radiography System with a Device for Monitoring X-ray Source-Detector Angle and Its Application in Chest Imaging

**DOI:** 10.3390/s17030531

**Published:** 2017-03-07

**Authors:** Tae-Hoon Kim, Dong-Woon Heo, Chang-Won Jeong, Jong-Hyun Ryu, Hong Young Jun, Seung-Jun Han, Taeuk Ha, Kwon-Ha Yoon

**Affiliations:** 1 Imaging Science Research Center, Wonkwang University, 460 Iksandeaero, Iksan, Jeonbuk 54538, Korea; tae_hoonkim@hanmail.net (T.-H.K.); dongwoon7069@naver.com (D.-W.H.); mediblue@wku.ac.kr (C.-W.J.); jhryu@wku.ac.kr (J.-H.R.); zip80@wku.ac.kr (H.Y.J.); mari153@nate.com (S.-J.H.); 10mari@hanmail.net (T.H.); 2 Department of Radiology, Wonkwang University School of Medicine, 460 Iksandeaero, Iksan, Jeonbuk 54538, Korea

**Keywords:** portable digital radiography (PDR) system, X-ray source-detector angle (SDA), radiography setting

## Abstract

This study developed a device measuring the X-ray source-detector angle (SDA) and evaluated the imaging performance for diagnosing chest images. The SDA device consisted of Arduino, an accelerometer and gyro sensor, and a Bluetooth module. The SDA values were compared with the values of a digital angle meter. The performance of the portable digital radiography (PDR) was evaluated using the signal-to-noise (SNR), contrast-to-noise ratio (CNR), spatial resolution, distortion and entrance surface dose (ESD). According to different angle degrees, five anatomical landmarks were assessed using a five-point scale. The mean SNR and CNR were 182.47 and 141.43. The spatial resolution and ESD were 3.17 lp/mm (157 μm) and 0.266 mGy. The angle values of the SDA device were not significantly difference as compared to those of the digital angle meter. In chest imaging, the SNR and CNR values were not significantly different according to the different angle degrees. The visibility scores of the border of the heart, the fifth rib and the scapula showed significant differences according to different angles (*p* < 0.05), whereas the scores of the clavicle and first rib were not significant. It is noticeable that the increase in the SDA degree was consistent with the increases of the distortion and visibility score. The proposed PDR with a SDA device would be useful for application in the clinical radiography setting according to the standard radiography guidelines.

## 1. Introduction

Digital radiography (DR) systems are frequently used in medical and industrial applications such as dental X-ray imaging, the aerospace industry, and in security and nondestructive testing [1]. Advancements in DR technologies have allowed more opportunities in the medical field because of a number of useful capabilities such as real-time acquisition, real-time post-processing, wireless techniques, and electronic data archiving and retrieval [2,3]. Such advantages of the DR system enhanced portability, mobility and smart environments due to the decreased size, light weight and better image quality depending on the improvement of the detector performance, as well as the implementation of multi-functional software. Among these advantages, mobility is an increasingly important topic in radiographic image acquisition [4]. Portable DR (PDR) detectors are suitable for intensive care units, emergency departments, outpatient clinics and so on [5]. Recent DR systems have increased the use of flat-panel detectors (FPDs) because they offer a lower radiation dose while preserving imaging quality as contrasted with thin-film transistor (TFT) detectors [6,7,8]. Therefore, it is important that newer DR systems are designed to solve the unmet needs of radiologists, technologists, administrators and private practices in conjunction with the advantages in DR technologies.

In clinical radiography, the X-ray source–to-detector distance (SDD) and X-ray source-detector angle (SDA) are important factors for acquiring appropriate image quality. According to standard clinical radiography guidelines [9,10], it is recommended for such imaging positions as follows: SDD of 180 cm, and SDA of 0° (around 5°, vertical incidence) for chest AP (anterior to posterior) and PA (posterior to anterior) imaging; 100 cm SDD and 15°–20° SDA (head side direction incidence) for cervical spine AP imaging; and 100 cm SDD and 5°–7° SDA (head side direction incidence) for lateral knee imaging. Currently, most commercial PDR systems are well equipped as a tape measure for SDD, whereas they are relatively less equipped a specific device for SDA compared to the equipment for SDD [11]. In clinical situations, unexpected conditions or extreme medical situations such as imaging experiments for immobilized bedridden patients, post-stroke hemiplegic patients and severely injured patients are often encountered [12]. From these viewpoints, a radiographic imaging system automatically determining the SDD and SDA can be helpful for clinical radiographic settings. However, there are few studies focusing on SDA measurement in clinical radiography.

Therefore, the purpose of this study was to develop a PDR system including a device for obtaining the SDA and to assess the imaging performance for the diagnosis of chest imaging.

## 2. Materials and Methods

### 2.1. Portable Digital Radiography System

**Figure 1:**
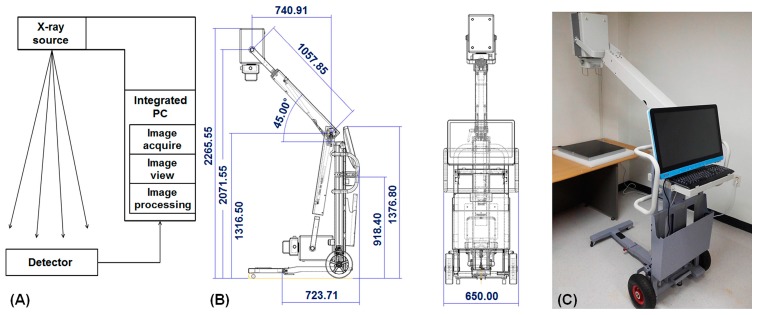
A simple diagram of portable digital radiography (PDR) (**A**), the detailed drawing of PDR structure (**B**) and developed PDR system (**C**).

The PDR main body consisted of an X-ray source, a FPD, and an integral PC (Figure 1A) and its detailed structure of PDR is shown in Figure 1B. The X-ray source generated 40–110 kVp and 20–100 mA with a focal spot size of 1.8 mm and weight of 20.4 kg (PXP-100CA, Poskom, Goyang, Korea). We used a high resolution amorphous silicon (a-Si)/CsI thin-film transistor (TFT) with PIN diode sensor-based FPD with 3072 × 3072 pixels (pixel pitch 140 × 140 μm), image size of 43 cm × 43 cm, and weight of 11 kg (FXRD-1717SA, Vieworks, Seoul, Korea). Image acquisition time of the FPD was below 1 s. The integrated PC for internal processing weighed 7.95 kg and used Windows 7 OS, an Intel Core i5 4300U with a 1.9 GHz CPU (POC-W242, Advantech, Taiwan). In order to increase flexibility and mobility of PDR system, X-ray source implemented two gas springs to smoothly move under a zero gravity state and PDR main body installed two main wheels and an auxiliary wheel. The main body of the PDR system attached on a slot for safekeeping an independent detector.

### 2.2. Internal Processing of Portable Digital Radiography

The integrated PC is detachable type and received image data from the FPD. In order to process received image data, the PDR system used a two-step correction algorithm as follows. In the first step, bad pixels on image data were detected with a 3 × 3 differentiation operator. When the differentiation at a given pixel was greater than a threshold value, the pixel was classified as a bad pixel. In the second step, the intensity value of the bad pixel was replaced by a bi-linear interpolating function of adjacent pixel values [13]. Then, the final image data were generated to Digital Imaging and Communications in Medicine (DICOM) file format. The DICOM files were stored in a specific directory on the PC hard disk storage.

### 2.3. Development and Performance Test of X-ray SDA Device

To determine the flip angle degree between an X-ray source and a detector, we developed an X-ray SDA device that consisted of a main board (Arduino Uno R3 board; ARDUINO, Ivrea, Italy), a 6 degrees of freedom (DOF) inertial measurement unit (IMU) shield (embedded in the ADXL345 accelerometer and the ITG-3200 gyro; Sparkfun Electronics, Boulder, CO, USA), and a Bluetooth module (HC-05 master, HC-06 slave; Shenzhen Rainbowsemi Electronics Co., Guangdong, China) [14]. This device was attached to the X-ray source (device 1) and detector (device 2), respectively. The Arduino main board was the core processing component coded by the Sketch software tool. The 6 DOF IMU shield included an accelerometer and gyro sensor, and transmitted the angle data to the Arduino through I2C communication after sensing the angle data from the X-ray source and detector. The 6 DOF is achieved by using a microelectromechanical system to sense translational movement in three perpendicular axes (surge, heave, sway) and rotational movement about three perpendicular axes (roll, pitch, yaw). The 6 DOF IMU designed to provide motion, position, and navigational sensing from a durable single device over 6 DOF (six-dimensional motion variants) [14]. The Bluetooth module was connected to the Arduino core processor.

**Figure 2:**
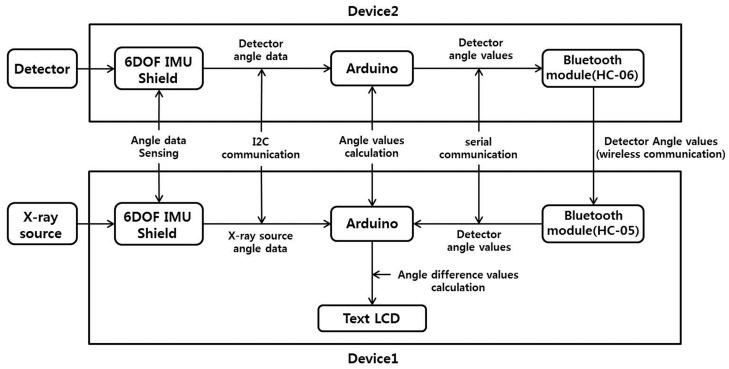
System block of the device for obtaining the X-ray source-detector angle (SDA).

Figure 2 shows the system block for processing procedures by the SDA device. The SDA value was measured as following steps. The Arduinos of devices 1 and 2 received angle data through I2C communication from the 6 DOF IMU Shield. Then, the Arduinos calculated the angle values for device 1 (X_S_ and Y_S_) and device 2 (X_D_ and Y_D_) by applying an algorithm to the angle data [15]. The Bluetooth module (HC-06) on device 2 received the angle values (X_D_ and Y_D_) via serial communication from the Arduino, and transmitted the angle values (X_D_ and Y_D_) to the Bluetooth module (HC-05) of device 1 in real-time. The Bluetooth module (HC-05) of device 1 transmitted the angle values (X_D_ and Y_D_) to the Arduino via serial communication. Finally, the Arduino of device 1 calculated the SDA difference (X_SDA_ and Y_SDA_) as follows: X_SDA_ = X_S_ − X_D_, Y_SDA_ = Y_S_ − Y_D_.

**Figure 3:**
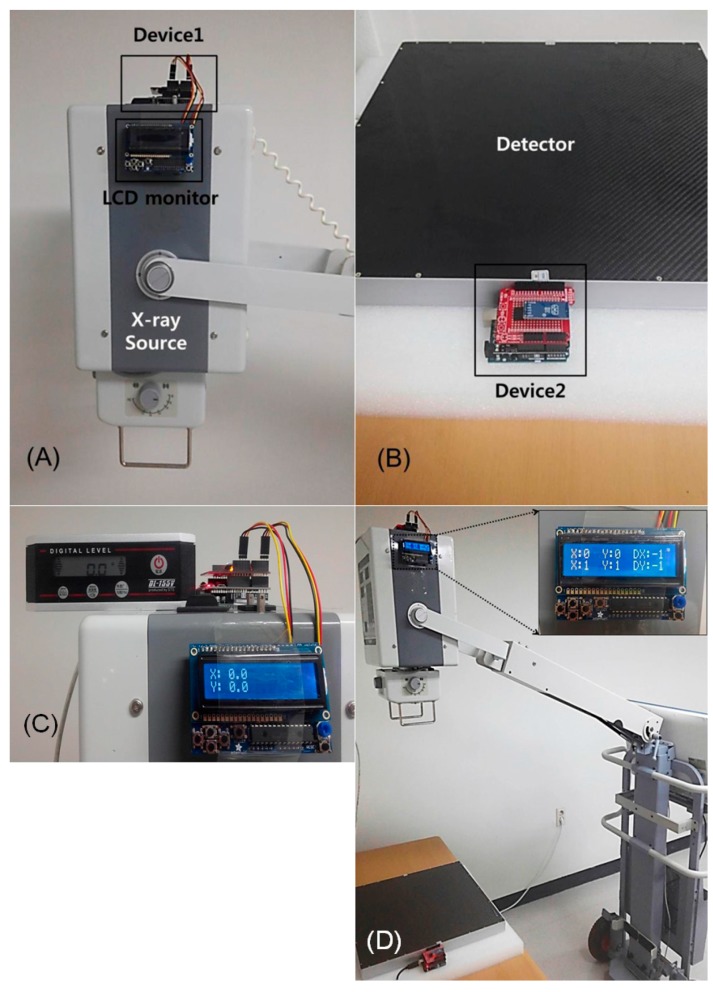
Details of the device for obtaining the SDA, which was attached on an X-ray source (**A**) and a detector (**B**). Photography showed the initial setting for obtaining the angle degree using the SDA device and digital angle meter (**C**). The ‘0.0°’ value on the SDA device was accordant with a ‘0.0°’ reference value on the digital angle meter. The measured angle degree using PDR with SDA device displayed on a LCD monitor (**D**).

In order to test the performance of SDA device, we used a digital angle meter (DL-155V, STS, Tokyo, Japan) with accuracies of 0.1%/degree (0°–10°/80°–90°), and 0.2%/degree (10°–80°) as a standard reference device. Figure 3 showed the developed SDA device attaching on the PDR system and the initial setting for achieving the angle degrees using both the SDA device and digital angle meter. First, the ‘0.0°’ value on the SDA device was accordant with a ‘0.0°’ reference value on the digital angle meter (Figure 3C). Next, the angle value of the digital angle meter changed from 0 to 60 degrees in units of five degrees, and then, the angle value on the SDA device measured three times. The measured angle degree using PDR with SDA device displayed on a LCD monitor (Figure 3D). Finally, the angle difference between digital angle meter and SDA device in each angle degree was calculated as mean difference of both angle values.

### 2.4. Measurements of Image Quality and Radiation Dose

The image quality of the PDR system was evaluated as signal-to-noise ratio (SNR), contrast-to-noise ratio (CNR), and spatial resolution using a bar phantom (X-ray test pattern type 18; FUNK, Berlin, Germany). The SNR and CNR were calculated as the ratio of the lead bar (0.05 mm thick) value to noise and the ratio of the lead bar-air contrast to noise, respectively [16]. Mean SNR and CNR values were obtained from six image sets using the developed PDR system. The modulation transfer function (MTF) has been used to evaluate spatial resolution of imaging systems [17]. This study was used a bar phantom to generate the MTF curve and was measured image resolution at 10% on MTF curve.

The radiation dose was calculated using the method described by the International Commission on Radiological Protection [18]. Entrance surface dose (ESD) is the absorbed dose including the contribution from backscatter [1]. The ESD measurement was performed using a dosimeter (Piranha, RTI Electronics, Mölndal, Sweden). This study was measured the ESD under the conditions of 80 kVp tube voltage, 4 mAs current, 100 ms and 1 m SDD.

### 2.5. Chest Radiography According to Different Angle Degrees

**Figure 4:**
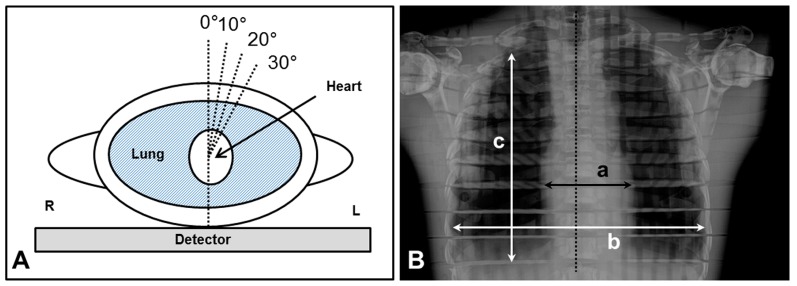
The angulation plane (**A**) and reference line setting (**B**) for the measurement of distortion on chest phantom: (a) cardiac transverse diameter (CTD); (b) thoracic transverse diameter (TTD); and (c) thoracic longitudinal diameter (TLD). L and R indicated left side and right side.

Chest radiographs were performed six times under the conditions of different angles using the developed PDR system with SDA device. To assess the image quality according to the angulation of the detector in the mediolateral plane of the patient, the angle value of the SDA changed from 0° (medial line) to 30° in units of 10 degrees (Figure 4). For the analysis of chest images, SNR and CNR according to different angle degrees were measured from 15 different points using the Alderson Radiation Therapy (ART) male phantom (ART-200A). The image distortion according to the changes of angulation was measured the deformity as percent changes in the sizes of cardiac transverse diameter (CTD), thoracic transverse diameter (TTD), and thoracic longitudinal diameter (TLD). Also, two expert radiologists (more than 10 years of experience) blindly evaluated each chest image, and reached a consensus regarding the anatomic landmarks [19]. The five anatomic landmarks were the border of the heart, clavicle, first rib, fifth rib and scapula. Each anatomic landmark on chest image data was analyzed according to the radiological diagnosis on a five-point scale: 1, definitely seen; 2, probably seen; 3, equivocal; 4, probably not seen; and 5, definitely not seen.

### 2.6. Statistical Analysis

All statistical analyses were performed using the Statistical Package for the Social Sciences (SPSS version 20.0, Chicago, IL, USA) software. Coefficient of variance (CV) of SNR and CNR was calculated for the variability of measurements in the PDR system [20]. The angle difference between both angle meters (SDA device and digital angle meter) was analyzed with the independent two sample *t*-test. According to the different angle degrees, the variation in image qualities, distortion and visibility scores were analyzed with the repeated-measures analysis of variance (rmANOVA) and Tukey’s post hoc tests. Two-sided *p*-values less than 0.05 were considered to indicate statistical significance.

## 3. Results

### 3.1. Development and Performances of PDR

**Figure 5:**
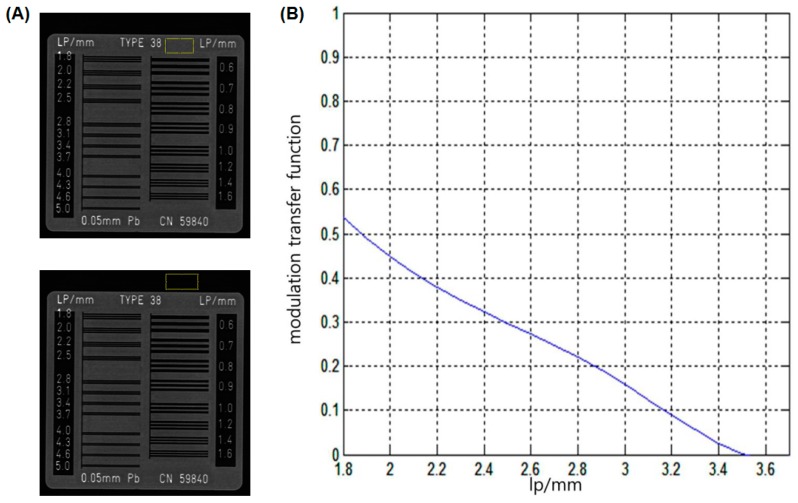
Bar phantom image obtained from the PDR to calculate the signal-to-noise ratio (SNR), contrast-to-noise ratio (CNR) and spatial resolution (**A**). Line-squares on the phantom images indicated the regions of interest for SNR and CNR measurements. A modulation transfer function (MTF, 10%) curve was used for resolution evaluation (**B**). The 10% MTF value is 3.17 lp/mm (157μm) on the image obtained from the PDR.

The size and weight of the PDR system were 723 mm (width) × 650 mm (depth) × 1376 mm (height) and approximately 60 kg, respectively (Figure 1). The PDR has a smooth flexibility of the X-ray source and can be moved easily by an adult. The mean SNR and CNR were 182.47 ± 6.75 (CV: 3.7%) and 141.43 ± 6.08 (4.3%), respectively. The overall CV values in the system were less than 5%. The spatial resolution at 10% MTF was 3.17 lp/mm (157 μm) (Figure 5) and the ESD was 0.266 mGy. Therefore, our PDR system is easily applicable for imaging bedside patients or inpatients such as immobilized bedridden patients and post-stroke hemiplegic patients.

### 3.2. Performance of the SDA Device

Figure 3 shows the features of the SDA device on the PDR system. The device was attached to the X-ray source and detector, respectively, and its size was 55 mm (width) × 80 mm (depth) × 35 mm (height). The angle values obtained from the SDA device were displayed in real time on the LCD monitor (1 times/s). The averaged angle values of the SDA device and digital angle meter are summarized in Table 1, and the mean difference in both angle values was 0.71° ± 0.15°. There was no significant angle difference between the digital angle meter and our SDA device (*p* > 0.05).

### 3.3. Chest Radiographic Study According to Different Angles for Clinical Application

**Figure 6:**
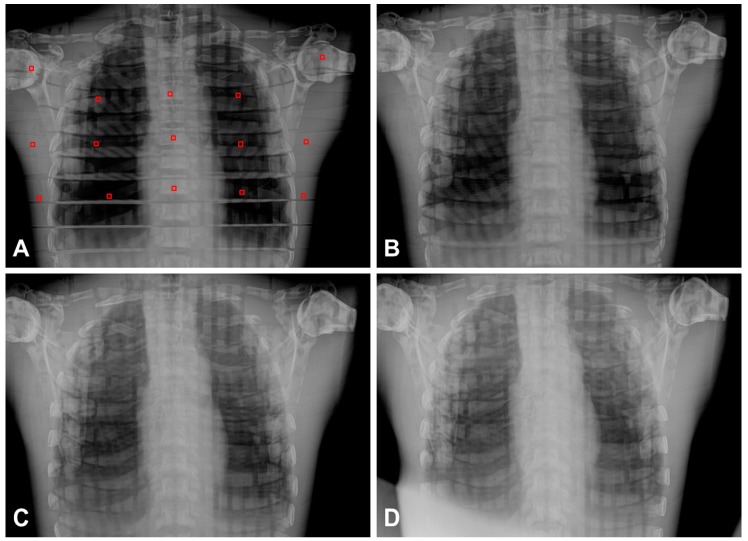
Representative chest AP images obtained from the developed PDR according to different angles: (**A**) 0° (=perpendicular); (**B**) 10°; (**C**) 20° and (**D**) 30° angle degree. The squares of red line (30 × 30 pixels) on AP image indicated 15 points for SNR and CNR measurements.

**Figure 7:**
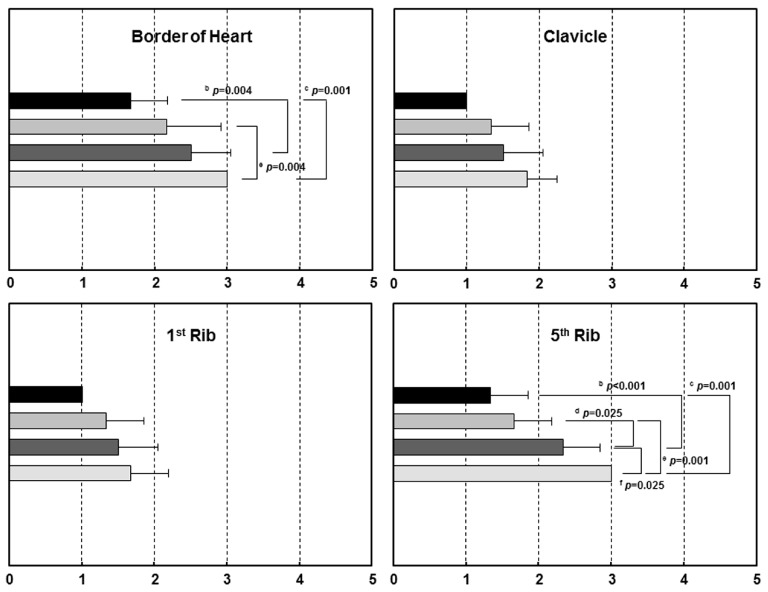

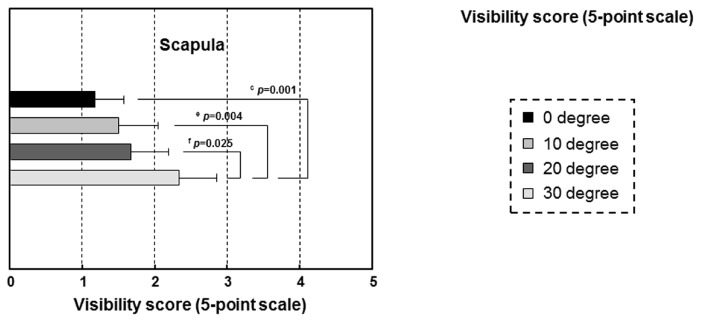
Mean anatomic landmark visibility scores according to different angle degrees. The significant difference between different angle degrees was analyzed with repeated-measures ANOVA and Tukey’s post hoc test: ^a^ 0 vs. 10; ^b^ 0 vs. 20; ^c^ 0 vs. 30; ^d^ 10 vs. 20; ^e^ 10 vs. 30; and ^f^ 20 vs. 30. Note that the scores including the border of the heart, the fifth rib and the scapula were significantly different from angle degrees (*p* < 0.05). Five-point scale: 1, definitely seen; 2, probably seen; 3, equivocal; 4, probably not seen; and 5, definitely not seen.

Figure 6 shows the representative chest AP images obtained from the developed PDR according to different angles. Imaging quality, distortion and visibility scores are summarized in Table 2. SNR and CNR values were not significantly different from different the angle degrees (rmANOVA, *p* > 0.05). The objective image qualities were not significantly different in this study; thus, it could be considered to be applicable for appropriate angle degrees in clinical experiments. However, the sizes and deformity (%) in the CTD, TTD and TLD increased according to the increment of angulation (rmANOVA, *p* < 0.001). The image distortion appeared more severe according to the increment of angulation. Figure 7 shows the mean visibility scores on anatomic landmarks according to different angle degrees. The visibility scores of the border of the heart, the fifth rib and the scapula were significantly different according to different angles (rmANOVA, *p* < 0.05), whereas the clavicle and first rib were not significant. It was noticeable that the increase in SDA degree was consistent with the increases of the distortion and visibility score.

## 4. Discussion and Conclusions

In this study, we successfully developed a PDR system with a SDA device. The current DR system was combined various information and communication technologies (ICT) that provide compact, portable, mobile, and smart environments for the diagnosis of patients [2,3]. The advantages of our PDR system include the increase of flexibility and mobility because the X-ray source implemented two gas springs to smoothly move under a zero-gravity state and the PDR main body installed two main wheels and an auxiliary wheel. Additionally, the slot attached on the PDR system is useful for safekeeping an FPD detector, and the integrated PC is easily detachable. Current PDR systems in radiography and radiological fields are necessary to provide images in the standard DICOM file format [1]. The proposed PDR system can save the acquired DICOM-formatted images to hard disk storage. Also, our SDA device can provide quantitative angulation information in clinical settings.

With regard to the imaging performance, the mean SNR and CNR values were 182 and 141, respectively. The spatial resolution of 10% MTF was 3.17 lp/mm (157 μm). The proposed PDR system had proper CV values (<5%) and image quality for clinical study. In terms of the radiation dose safety, the reduction of the radiation dose for patients is an important issue. This emphasizes the importance of the as low as reasonably achievable (ALARA) concept. At present, cancer induction is a stochastic risk with a linear-no-threshold dose model; therefore, reduced exposure to radiation decreases the risk of the development of cancer, such as leukemia, in subjects [21]. To resolve this, the ESD must be reduced while maintaining the image quality. According to the recommendation of the radiographic guideline [18], the ESD limit for the chest is 0.4 mGy. The guidelines of the European Union (EU) [22] and the American College of Radiologists (ACR) [23,24] suggest that the mean entrance surface exposure should range from 0.05 to 0.3 mGy per exposure scan in newborns, infants and children. In the present study, the ESD of the proposed PDR was 0.266 mGy under the conditions at 80 kV, 0.4 mA, 100 ms and 1 m SDD. Therefore, the proposed PDR system could be likely to be safe in clinical radiography settings.

The accuracy test of the developed angle device is essential to evaluate the performance of the SDA device. From this viewpoint, we used a digital angle meter with an accuracy of 0.1%/degree (0°–10°/80°–90°) and 0.2%/degree (10°–80°) as a reference device. In the present study, the angle difference between both the digital angle meter and the SDA device was below 1° and there was no significant difference. Therefore, the SDA device provided accurate SDA values to the operator and the angle values will be useful for the clinical radiography setting. Moreover, due to our SDA device being attached easily to any other systems, it can be widely used with the products requiring accurate angle values. The current accuracy of our SDA device is good enough (<0.8°) for chest imaging, but its accuracy needs improvement for more demanding imaging such as the computed tomography (CT) imaging. For clinical applications in unexpected conditions or extreme medical situations, it should be considered that the increment of angulation leads to image distortion of the anatomy.

With regard to the factors for clinical imaging, SDD and SDA are important factors for acquiring appropriate image quality according to standard clinical radiography guidelines [9,10]. From these radiographic guidelines, it is recommended the imaging positions as cervical spine AP imaging of 100 cm SDD and 15°–20° SDA (head side direction incidence), and lateral knee imaging SDD of 100 cm and SDA of 5°–7° (head side direction incidence), respectively. Especially, for PA and AP chest radiography (SDD of 180 cm), it is common practice to have a small caudal tube angulation (SDA around five degrees). We believed to reduce the dose to the radiosensitive thyroid gland. Also, the proposed PDR system with the SDA device could provide both SDD and SDA values, and thus it might be considered to be applicable for the measurements at any distance and angle degree of the imaging positioning under unexpected conditions or in extreme medical situations. In the future, device development for the real-time tracking of SDD would be helpful for immobilized bedridden patients, post-stroke patients, severely injured patients, etc.

In conclusion, the proposed PDR system provided a flexible structure, light weight, and high portability, as well as accurate SDA and SDD values according to the clinical radiography standard guidelines. This system will be useful for applications in clinical radiography.

## Figures and Tables

**Table 1:** Angle values and angle difference of digital angle meter and developed X-ray source-detector angle (SDA) device.

Degree (°)	Digital Angle Meter (A, Mean ± SD)	Developed SDA Device (B, Mean ± SD)	Angle Difference (B-A)
0	0.00 ± 0.00	0.00 ± 0.00	0.00
5	5.00 ± 0.00	5.30 ± 0.08	0.30
10	10.00 ± 0.00	10.70 ± 0.08	0.70
15	15.00 ± 0.00	15.50 ± 0.08	0.50
20	20.00 ± 0.00	20.87 ± 0.04	0.87
25	25.00 ± 0.00	25.80 ± 0.08	0.80
30	30.00 ± 0.00	30.70 ± 0.08	0.70
35	35.00 ± 0.00	35.77 ± 0.04	0.77
40	40.00 ± 0.00	40.77 ± 0.12	0.77
45	45.00 ± 0.00	45.77 ± 0.04	0.77
50	50.00 ± 0.00	50.80 ± 0.08	0.80
55	55.00 ± 0.00	55.77 ± 0.04	0.77
60	60.00 ± 0.00	60.73 ± 0.04	0.73
*p*-value *	0.865		0.71 ± 0.15

Abbreviation: SD, standard deviation; SDA, source-detector angle. Data are presented as mean ± SD after three measurements. Angle difference between digital angle meter (A) and developed device (B) in each degree was calculated as mean (B-A). * The difference between angle values of digital angle meter (A) and developed device (B) was analyzed with the independent two-sample *t*-test.

**Table 2:** SNR, CNR, distortion and visibility values on chest AP images according to different angles.

	Angle Degree	0°	10°	20°	30°	*p*-Value *
Value						
SNR		408.7 ± 160.0	408.8 ± 157.8	428.0 ± 176.3	463.4 ± 155.9	0.082
CNR		82.3 ± 23.0	100.0 ± 30.9	102.8 ± 33.1	98.2 ± 28.8	0.114
Distortion ^†^						
CTD (size, mm)		101.9 ± 0.3	104.4 ± 0.3	106.6 ± 0.4	107.5 ± 0.3	<0.001 ^abcdef^
(deformity, %)		(100.0%)	(102.5%)	(104.6%)	(105.5%)	
TTD (mm)		278.0 ± 0.3	278.5 ± 0.1	278.7 ± 0.1	279.4 ± 0.3	<0.001 ^abcdef^
(%)		(100.0%)	(100.2%)	(100.3%)	(100.5%)	
TLD (mm)		250.2 ± 1.0	260.7 ± 1.3	266.3 ± 1.3	273.7 ± 1.4	<0.001 ^abcdef^
(%)		(100.0%)	(104.2%)	(106.4%)	(109.4%)	
Visibility ^‡^						
Border of Heart		1.67 ± 0.52	2.17 ± 0.75	2.50 ± 0.55	3.00 ± 0.00	0.044 ^bce^
Clavicle		1.00 ± 0.00	1.33 ± 0.52	1.50 ± 0.55	1.83 ± 0.41	0.110
1st Rib (Rt-side)		1.00 ± 0.00	1.33 ± 0.52	1.50 ± 0.55	1.67 ± 0.52	0.292
5th Rib (Rt-side)		1.33 ± 0.52	1.67 ± 0.52	2.33 ± 0.52	3.00 ± 0.00	<0.001 ^bcdef^
Scapula		1.17 ± 0.41	1.50 ± 0.55	1.67 ± 0.52	2.33 ± 0.52	0.032 ^cef^

Abbreviation: CNR, contrast-to-noise ratio; CTD, cardiac transverse diameter; SNR, signal-to-noise ratio; TTD, thoracic transverse diameter; TLD thoracic longitudinal diameter. Data are presented as mean ± SD after six measurements at each point. * The significant difference between different angle degrees was analyzed with repeated-measures ANOVA with Tukey’s post hoc test: ^a^ 0 vs. 10; ^b^ 0 vs. 20; ^c^ 0 vs. 30; ^d^ 10 vs. 20; ^e^ 10 vs. 30; and ^f^ 20 vs. 30. ^†^ The image distortion according to the changes of angulation was calculated the deformity as the percent changes in the sizes of the diameter. The statistical analysis was used with repeated-measures ANOVA and Tukey’s post hoc test as follows: ^a^ 0 vs. 10; ^b^ 0 vs. 20; ^c^ 0 vs. 30; ^d^ 10 vs. 20; ^e^ 10 vs. 30; and ^f^ 20 vs. 30. ^‡^ The value of each anatomical landmark on the chest image was analyzed according to the radiological diagnosis on a five-point scale: 1, definitely seen; 2, probably seen; 3, equivocal; 4, probably not seen; and 5, definitely not seen.

## Abbreviations

The following abbreviations are used in this manuscript:

ALARA	as low as reasonably achievable
CNR	contrast-to noise
CTD	cardiac transverse diameter
CV	coefficient of variance
DICOM	Digital Imaging and Communications in Medicine
DOF	degree of freedom
DR	digital radiography
ESD	entrance surface dose
FPD	flat-panel detector ratio
ICT	information and communication technologies
IMU	inertial measurement unit
MTF	modulation transfer function
PDR	portable digital radiography
rmANOVA	repeated-measures analysis of variance
SDA	X-ray source-detector angle
SDD	X-ray source-detector distance
SNR	signal-to-noise ratio
TFT	thin-film transistor
TLD	thoracic longitudinal diameter
TTD	thoracic transverse diameter
